# Reasoning beyond clicks: Disentangling counterexamples and probabilities in conditional reasoning

**DOI:** 10.3758/s13421-025-01796-9

**Published:** 2025-11-04

**Authors:** Markus Knauff, Lupita Estefania Gazzo Castañeda

**Affiliations:** Experimental Psychology and Cognitive Science, University of Giessen, Otto-Behaghel-Str. 10F, 35394 Giessen, Germany

**Keywords:** Reasoning, Methodology, Mental models, Deduction, Uncertain reasoning, Probabilities

## Abstract

In reasoning experiments, participants usually evaluate conclusions by clicking on predefined response alternatives or rating scales. However, such response formats may elicit specific cognitive reasoning strategies. To avoid such biases, we employed an open response format, allowing participants to formulate and explain their conclusions freely in complete sentences. Across two experiments, participants generated over 1,300 written responses, which we categorized as *certain, uncertain based on counterexamples, *or* uncertain based on probabilities*, using a predefined coding scheme. Participants frequently provided certain responses rather than expressing graded degrees of belief. When uncertainty was expressed, it was more often justified by concrete counterexamples than by probabilistic reasoning. We introduce the term *probabilistic masking* to describe the phenomenon whereby graded response formats may suppress the consideration of certain inferences or counterexamples, thereby biasing empirical accounts of human inference toward probabilistic interpretations.

**Supplementary Information:**

The online version contains supplementary material available at 10.3758/s13421-025-01796-9.

## Introduction

For decades, research on human reasoning focused primarily on deductive inferences (Evans, Newstead, & Byrne, 1993; Johnson-Laird & Byrne, 1991; Knauff & Spohn, 2021). Such inferences provide insight into the structure of logical thought and the norms of formal reasoning. However, over the past two decades, researchers have shifted attention away from this kind of human reasoning. One reason is that the underlying cognitive process are now considered relatively well understood (e.g., Evans, 2021; Johnson-Laird, 2010). The other is that deductive reasoning captures only a narrow slice of human cognition. Everyday reasoning often involves uncertainty, incomplete information, and varying degrees of belief – features that are better captured by non-deductive forms of inference (Hahn & Oaksford, 2007; Oaksford & Chater, 2007). The key question now is: Why are people often uncertain about the validity of a conclusion? 

One possible explanation is that they can think of *counterexamples* to a deductively valid conclusion, leading them to reject it. Another possibility is that they interpret the premises *probabilistically*, which prevents them from arriving at a definite conclusion.

In numerous experiments conducted in our laboratory, we have demonstrated that the way people reason and the conclusions they draw depend largely on how the task is framed and which response options are provided (Gazzo Castañeda & Knauff, 2016a, 2016b, 2018, 2019, 2021a, 2021b). These findings motivated the present study: We aimed to minimize response constraints and to give participants as much freedom as possible in formulating their conclusions. To reach this goal, we deliberately avoided predefined response options and rating scales, which have become common in reasoning research in recent years.

Despite its methodological focus, the study also builds on careful theoretical considerations. Here we briefly introduce the two dominant theories of human reasoning: the theory of mental models and probabilistic reasoning theories. What both theories have in common is the assumption that people rely on their prior knowledge, which leads to uncertainty about whether a conclusion is certainly (deductively) valid. However, the key difference is that the mental model theory (Hinterecker, Knauff, & Johnson-Laird, 2016; Johnson-Laird, Khemlani, & Goodwin, 2015; Johnson-Laird & Ragni, 2019) suggests that people generate mental representations of possibilities and search for counterexamples, while probabilistic theories argue that people assess the likelihood of premises and conclusions based on prior knowledge (Oaksford & Chater, 2007, 2020). The next section briefly summarizes the key differences between these two theoretical frameworks.

## Prior knowledge, counterexamples, and probabilities

Consider the following conditional inference:

- If the traffic light is red, then the car stops.
- The traffic light is red.
- Does the car stop?

According to classical logic, the correct answer is “yes”: if *p* then *q*; *p*; therefore *q* – a logically valid inference called *Modus Ponens* (MP). But, in daily life, people often refrain from this logically valid conclusion (e.g., Oaksford & Chater, 1995; Pollock, 1987; Stenning & van Lambalgen, 2005). They know that in some cases cars do not stop at a red traffic light. The less a person believes in a conclusion, the less it is accepted.

Degrees of beliefs can also affect invalid inferences. For example:

- If the traffic light is red, then the car stops.
- The car stops.
- Is the traffic light red?

Individuals often respond to this inference with a “yes,” although it is an incorrect response according to classical logic. The inference is called *Affirmation of the Consequent* (AC): “if p then q; q; therefore p.” AC is invalid because when q is true (e.g., the car stops), p can be true or false (e.g., the car can also stop for a pedestrian crossing or due to having no fuel). Nothing follows logically from the premises. Yet, participants may respond correctly that no conclusive inference can be made if they consider situations in which q occurs independently of p (e.g., the car stops at a stop sign, a crossing, in a traffic jam, etc.).

But how exactly do people form these degrees of beliefs? Mental model theory posits that individuals construct internal representations of possible scenarios based on the given premises (Johnson-Laird et al., 2015; Johnson-Laird & Ragni, 2025). When faced with a deductively valid conclusion, they may nonetheless reject it if they can think of at least one plausible counterexample. In the example above, a person might recall instances in which cars failed to stop at a red light – due to mechanical failure, driver distraction, or deliberate violation of the rule. Such counterexamples introduce uncertainty, even when the inference is logically valid.

In contrast, probabilistic reasoning theories (e.g., Oaksford & Chater, 2007, 2020) propose that reasoning is grounded in the subjective probabilities assigned to premises and conclusions. Rather than searching for counterexamples, individuals assess the strength of premises based on prior experience and general knowledge. In the traffic light example, one might estimate how often cars actually stop when the light is red. If the estimated rate is 90%, the conclusion may be judged as highly probable but not certain. This approach aligns with Bayesian models of reasoning, which describe belief updating in light of new information (Oaksford & Chater, 2007, 2020; Oaksford et al., 2000).

Mental model theory has been criticized for its lack of clarity about how counterexamples are retrieved, represented, and weighed during reasoning. While the search for counterexamples is central to the theory, how people generate them in practice and how they judge which counterexamples are strong or relevant enough to reject a conclusion remain undetermined (Oaksford & Chater, 2020; Over, 2009).

Probabilistic theories have also faced criticism. For example, Khemlani et al. (2015) asked participants to estimate three probabilities: pr(P), pr(Q), and pr(If P then Q). The results showed that participants often gave inconsistent estimates, with even greater variability in cases involving conjunctions and disjunctions. Similarly, Hinterecker et al. (2016) found that when participants assessed probabilities based on disjunctive premises, their combined estimates frequently exceeded 100%, suggesting cognitive overload or conceptual confusion.

A key limitation of most current studies is that they employ widely varying methods to elicit reasoning responses. In particular, some tasks prompt uncertainty and graded responses (e.g., likely/unlikely), potentially favoring one type of reasoning over another. Such methodological differences make it difficult to compare findings and to draw general conclusions about how people reason. Moreover, predefined response formats may constrain participants’ thinking or obscure the reasoning strategies they would naturally apply.

To address these limitations, the present study adopts an open-ended format that allows participants to express their conclusions in their own words. This design is intended to reveal whether people spontaneously rely on counterexamples, probabilistic assessments, or other strategies when evaluating conditionals under uncertainty. Specifically, our hypotheses were as follows:

- H1: Experimental task formats can systematically shape participants’ reasoning, producing behavioral artifacts that make them appear more “probabilistic” than they actually are. When free to respond, participants generate categorical (deductive) inferences more often than probabilistic theories predict.
- H2: Even when participants are uncertain about a conclusion, experimental formats may bias them toward probabilistic responses. However, when given more freedom, participants are more likely to generate counterexamples to express uncertainty than to rely on explicit probabilistic information.

## Our study

We conducted two experiments to test these hypotheses. The core idea was straightforward: we wanted to give participants maximal freedom in the way in which they responded and explained their reasoning – without prescribing specific response formats or instructing them on how to think. In short, our goal was to observe human reasoning as spontaneously and naturally as possible.

While this approach was convenient for participants, it posed methodological challenges for us as researchers. How should we analyse the open responses? What counts as a conclusion based on counterexamples? Which conclusions rely on subjective probabilities? To address these questions, we invested significant effort into developing a detailed classification scheme that assigns each response to a specific reasoning category. In the sections that follow, we first introduce this classification scheme and describe how it was developed. We then present a pilot study used to identify conditionals with high and low prior probabilities – necessary for designing the main experiments. Then we outline the two experiments in detail. Finally, we highlight the theoretical implications of our findings, and suggest directions for future research.

## Classification scheme

Our first task was to predefine a classification scheme for categorizing participants’ responses in the experiments. For this, we defined four distinct categories:

1. Certain responses
2. Uncertain responses based on counterexamples
3. Uncertain responses based on probabilities
4. Unusable responses.

Category 4 included responses that were nonsensical, showed a fundamental misunderstanding of the instructions, or could not be meaningfully analyzed. In Experiment 1, 15 responses fell into this category; in Experiment 2, 19 did. These responses were excluded from further analysis.

The remaining three categories we defined as follows:

- Category 1: Responses that consisted of a simple “yes” or “no” without elaboration, or statements that merely reiterated p, q, or their negations without further specification (e.g., “The car will stop”).
- Category 2: Responses that included concrete counterexamples indicating exceptions to the conditional rule (e.g., “Not if the driver overlooks the traffic light, escapes from the police, etc.”).
- Category 3: Responses that used probabilistic expressions such as probably, maybe, perhaps, not always, sometimes, frequently, etc., but did not include counterexamples (e.g., “Usually yes, but it is possible that the driver will not stop”).

Two independent student assistants, who were unfamiliar with existing theories of reasoning and held no specific theoretical commitments, classified more than 1,300 participant responses. Inter-rater reliability – defined as the proportion of responses assigned to the same category by both raters – was 97.29% in Experiment 1 and 98.31% in Experiment 2. These values indicate a high level of consistency and suggest that the classification scheme was both clear and robust. In the few cases of disagreement, the raters discussed their decisions and resolved all discrepancies through consensus.

## Pilot study

Our next task was to carry out a pilot study to identify conditionals with low and high probabilities. We constructed a pool of 48 conditionals and asked 52 participants to rate the conditional probabilities P(p|q) and P(q|p) on a scale from 0% to 100%. Some conditionals were newly created, while others were taken from previous studies (De Neys et al., 2002; Gazzo Castañeda & Knauff, 2020; Verschueren et al., 2005). Here is an example of a problem from the pilot study that required estimating P(p|q) or P(q|p):
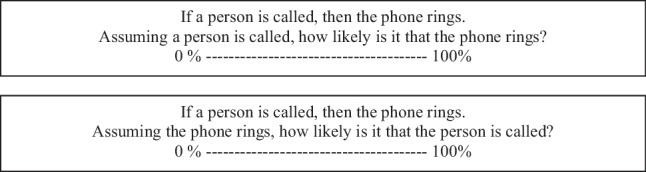


Based on these subjective probability ratings, we selected 12 conditionals for the main experiment: six with high conditional probabilities and six with low conditional probabilities. 

The high-probability items had an average P(q|p) of 87.66% (SD = 6.80) and an average P(p|q) of 88.75% (SD = 4.37). The low-probability items had an average P(q|p) of 54.46% (SD = 4.92) and an average P(p|q) of 51.20% (SD = 7.94). The full list of conditionals is presented in Table 1.

**Table 1:** Selected conditionals from the prior study together with their conditional probabilities

Conditional	Conditional probabilities *M* (*SD*)	
	P(*q*|*p*)	P(*p*|*q*)
*High conditional probabilities*		
If a person has touched a glass with their bare hands, then their fingerprints will be on the glass.	.96 (.09)	.91 (.18)
If a person receives a call, then their phone will ring.	.85 (.17)	.85 (.22)
If a person is looking for a job, then they write applications.	.81 (.17)	.86 (.19)
If a person exercises, then they build muscle	.81 (.18)	.84 (.14)
If the light switch is turned on, then the light will come on.	.89 (.16)	.92 (.15)
If water is heated to 100 °C, then it boils.*	.95 (.17)	.95 (.17)
*Low conditional probabilities*		
If a person suppresses their sneeze, then blood vessels in the eye burst.	.53 (.32)	.45 (.26)
If a person eats a lot of sweets, then they will develop diabetes.	.52 (.30)	.45 (.29)
If a person exercises, then they will lose weight.	.56 (.24)	.51 (.21)
If a person eats a lot of vegetables, then they are healthy.	.63 (.23)	.61 (.18)
If a person drinks alcohol, then they have difficulty remembering the next day.	.49 (.25)	.43 (.22)
If a person snacks a lot, then they are overweight.	.55 (.20)	.60 (.21)

The last high-probability conditional is marked with an asterisk (*), as one reviewer pointed out that, unlike the other conditionals, this item contains a mass noun, whereas all others involve count nouns, which could have influenced participants’ interpretations. In response, we recalculated all subsequent analyses, excluding this particular item. Since the results showed no significant differences between analyses with and without this item, we decided to retain it. Thus, the 12 conditionals listed in Table 2 were used in the following experiments.

## Experiment 1

### Methods

#### Participants

Twenty participants took part in the reasoning experiment (15 female, five male). Their mean age was 23.85 years (*SD* = 2.03). Participants were recruited via social networks. Participants with a psychological background were excluded due to possible prior knowledge on reasoning problems. All gave informed consent.

#### Materials, design, and procedure

The six conditionals with high and six with low conditional probabilities from the pilot experiment were embedded in MP and AC inferences, resulting in 24 inferences in total. Thus, the experiment followed a 2 × 2 within-subjects design, with conditional probability (high vs. low) and inference type (MP vs. AC) as factors. The dependent variable was the percentage of responses classified according to our classification system for each experimental condition.

The experiment was conducted online using SocSci Survey (Leiner, 2021). Participants were informed that they would be presented with 24 problems, each consisting of an if-then rule followed by a question asking for a conclusion. The instruction was:

*You will be presented with 24 tasks consisting of an if-then statement, a fact and a question about a conclusion. Your task is to read these carefully and write your conclusion in the text field below. Please answer as you would in everyday life. You are free to formulate your answer as you wish. There are no right or wrong answers.*

All problems were presented in a random order after a short practice trial consisting of one MP and one AC inference. Here is an example of an MP problem:

If a person is called, then the phone rings.

A person is called.

Does the phone ring?
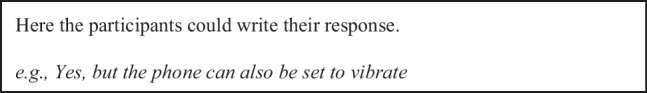


Participants took approximately 30 min to complete the experiment.

### Results

The first column of Table 2 shows the percentage distribution of responses falling in the three categories. About one-third of the responses were certain, another third expressed uncertainty in terms of probability, and approximately 40% used counterexamples to explain uncertainty. Since no reliable differences were found between the MP (category 1: 30.88%; category 2: 44.96%; category 3: 24.17%) and AC (category 1: 28.29%; category 2: 38.79%; category 3: 32.92%) conditions, the results for these conditions were pooled. Although the following comparisons are not orthogonal, the differences between certain and uncertain responses (using counterexamples or probabilities) were significant (Wilcoxon test: z = 3.58, p <.001). Similarly, there was a clear difference between uncertain responses using counterexamples and those using probabilistic terms, although this difference reached the level of significance only for high-probability problems (Wilcoxon test: z = 2.09, p =.036). Overall, the type of response depended on conditional probability. As shown in columns 2 and 3 of Table 1, participants were significantly more likely to express certainty in high-probability problems than in low-probability problems (Wilcoxon test: z = 3.35, p <.001), and they used probabilistic expressions more often in low-probability problems compared to high-probability problems (Wilcoxon test: z = 2.99, p =.003). For illustration, some of the participants’ written answers are presented in Table 3.

**Table 2:** Relative frequency of answers generated by participants in Experiment 1 [in %]

	All	Low probability	High probability
Certain	29.6	13.9	45.3
Uncertain with counterexamples	41.9	45.6	38.0
Uncertain with probabilities	28.54	40.33	16.6

### Discussion

To our knowledge, this is the first experiment on conditional reasoning in which participants were free to express their conclusions and justifications in their own words. The most surprising result is that in about one-third of the items, participants reported being certain about their inferences. This finding supports our first hypothesis that the uncertainty typically reported in the current literature may, at least in part, be an artefact of the response formats commonly used in reasoning studies. Widely used rating scales may subtly prompt participants to express degrees of uncertainty, even when they would not do so spontaneously. In contrast, the open-ended format employed in Experiment 1 allowed for more natural and unprompted responses – raising the possibility that previous studies may have underestimated the prevalence of confident, deductive reasoning.

This result also challenges a central assumption of probabilistic theories of reasoning – namely, that people generally interpret conditionals in terms of uncertainty rather than certainty (cf. Oaksford & Chater, 2007, 2020). Oaksford (2015) even suggests that what appear to be deductive inferences are in fact probabilistic inferences involving extreme probabilities. Our data call this assumption into question by showing that people can and do arrive at conditional conclusions with a high degree of confidence.

A second key finding concerns the type of uncertainty participants expressed when they were unsure. Their reasoning more often involved concrete counterexamples than references to probabilities or likelihoods. Instead of assigning a degree of probability to the conclusion, participants either rejected it by citing specific counterexamples, or they accepted it while pointing out exceptional cases in which it would not apply. This supports the core claim of mental model theory that people evaluate conditionals by searching for counterexamples rather than by using probabilities.

Overall, the observed reliance on counterexamples supports our second hypothesis and raises challenges for probabilistic models of reasoning. It may suggest that such models underestimate the cognitive salience of case-based reasoning, leading to probabilistic assessments relying on qualitative evaluations of plausibility. We return to this point in the *General discussion.*

## Experiment 2

The high number of certain responses observed in Experiment 1 suggests that commonly used rating scales may implicitly prompt participants to express uncertainty – raising the possibility that prior findings have underestimated the prevalence of categorical reasoning. However, we must also consider the reverse possibility: that our own method may have biased participants toward certainty. Previous research has shown that explicitly presenting a conditional rule as the major premise of an argument can prime a logical interpretation and increase conclusion endorsement (Klauer et al., 2010). In other words, when participants are cued to treat a task as a logical inference problem, they may be more likely to respond in a binary, deductive fashion.

To address this concern, Experiment 2 modified the task structure. Rather than presenting inference problems in formal conditional form (e.g., “If p, then q”), we refrained from explicitly stating the conditional rule. This reduced the salience of the logical structure and allowed us to examine whether participants would still respond with confidence – or instead show greater uncertainty and rely more on probabilistic reasoning. Experiment 2 thus provides a critical test of the generalizability of the findings from Experiment 1.

### Methods

#### Participants

Thirty-seven participants took part in the experiment (25 female, 12 male). Their mean age was 24.73 years (*SD* = 3.60). Again, participants were recruited via social networks and participants with a psychological background were excluded due to possible prior knowledge on reasoning problems. All gave informed consent.

#### Materials, design, and procedure

The materials, design, and procedure were identical to those used in Experiment 1, with one key difference: in Experiment 2, the conditional premise was no longer explicitly presented in the inference tasks. For example:

A person is called.

Does the phone ring?
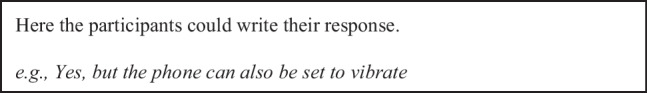


### Results

The first column of Table 3 shows the percentage distribution of responses across the three categories. As in Experiment 1, approximately one-third of the responses were classified as certain, around 23% expressed uncertainty using probabilistic terms, and nearly half conveyed uncertainty through counterexamples. Again, no reliable differences emerged between the MP (Category 1: 28.02%; Category 2: 45.56%; Category 3: 26.42%) and AC conditions (Category 1: 33.33%; Category 2: 46.17%; Category 3: 20.50%), so we pooled the data across these conditions.

Overall, the response pattern was similar to that observed in Experiment 1, but more pronounced. First, the difference between certain and uncertain responses (i.e., those based on counterexamples or probabilistic terms) remained significant (Wilcoxon test: z = 3.61, p <.001). Second, participants again gave significantly more uncertain responses based on counterexamples than on probabilistic expressions. While this effect had reached significance only in high-probability problems in Experiment 1, it was now observed across all problem types (Wilcoxon test: z = 2.94, p =.003).

As before, the type of response was modulated by conditional probability: participants were significantly more likely to express certainty in high-probability problems than in low-probability ones (Wilcoxon test: z = 5.16, p <.001). Conversely, the use of probabilistic expressions to indicate uncertainty was significantly more frequent in low-probability problems than in high-probability ones (Wilcoxon test: z = 4.63, p <.001).

**Table 3:** Relative frequency (in %) of answers generated by participants in Experiment 2

	All	Low probability	High probability
Certain	30.7	17.1	44.2
Uncertain with counterexamples	45.9	48.00	43.7
Uncertain with probabilities	23.5	34.9	12.0

### Discussion

In Experiment 2, we removed the explicit formulation of the conditional rule to reduce the salience of logical structure. Interestingly, this change did not diminish the frequency of confident responses. On the contrary, the pattern observed in Experiment 1 became even more pronounced. Once again, approximately one-third of the responses were classified as certain, despite the absence of any formal logical cue.

More importantly, among the uncertain responses, participants continued to rely more frequently on counterexamples than on probabilistic expressions. This effect was not only replicated but also amplified: while in Experiment 1 it reached significance only for high-probability items, in Experiment 2 it was observed across all types of problems.

A particularly noteworthy finding is that counterexample-based reasoning was most prevalent in response to high-probability conditionals. This may appear counterintuitive at first. Why would participants be more likely to imagine exceptions precisely when a conditional seems likely to be true? A possible explanation is that high-probability conditionals activate strong expectations, which in turn invite reflection on exceptional cases. That is, when something is generally expected to occur, the mind may become more attuned to conditions under which it might not. This interpretation aligns with the mental model theory’s view that counterexamples play a central role in how people assess the strength or reliability of a conclusion (Johnson-Laird, 2006).

At the same time, the data speak against the idea that participants naturally assess conditionals in terms of abstract probabilities. While some probabilistic reasoning did occur – especially in response to low-probability conditionals – it was the less frequent mode of expressing uncertainty. This again casts doubt on the descriptive adequacy of probabilistic models when applied to spontaneous, unconstrained reasoning. Before returning to the broader theoretical implications of these findings, it is worthwhile to take a closer look at some of the actual responses participants provided.

#### Examples of participants’ answers

As already mentioned, across both experiments we collected and categorized over 1,300 freely generated responses. While the main quantitative results have been presented above, we now provide a few illustrative examples. These responses were not subject to further systematic analysis and are not intended to be exhaustive or statistically representative. However, we include a selection in the Appendix to give readers a clearer impression of the range and character of participants’ reasoning. For illustration, we selected only a small number of responses. We did not include the certain responses – which made up approximately 30% of the data – as they were typically brief and uninformative (e.g., “Yes,” “Obviously,” “Correct,” or “Wrong”). Instead, we focused on uncertain responses, particularly those that reflect the use of counterexamples or probabilistic considerations. Examples were drawn from two tasks in both experiments, as we did not observe any systematic differences between responses with and without explicit mention of the conditional rule.

One of the most striking observations is that the well-known *suppression effect* (Byrne, 1989) clearly manifests in participants’ responses. In several problems where a logically valid inference (MP) could have been drawn, participants were uncertain about it – often explicitly referring to counterexamples that would prevent the conclusion from holding. For example, when asked whether a phone rings if a person is called, several participants responded: “It could be on silent,” or “It might be in flight mode.” These responses illustrate how prior knowledge and the recognition of exceptions can make a logically valid inference questionable, leading to more uncertainty in the inference.

Interestingly, many explanations also demonstrate an intuitive grasp of the invalidity of AC inferences. Rather than affirming the inference outright, they often pointed out that the consequent could occur independently of the antecedent. For instance, in response to the conditional “If a person exercises, they lose weight,” participants noted that someone might lose weight “due to illness,” or “because of a diet,” not necessarily due to physical activity. Such responses suggest a widespread sensitivity to the possibility of alternative causes – in particular in an open-ended response format probably triggering more reflective thinking (see below).

A third class of responses expressed uncertainty in probabilistic terms, without reference to specific counterexamples. Here, participants employed expressions such as “probably,” “maybe,” or “could be” – suggesting a graded interpretation of the conditional rather than a categorical one. However, only two participants gave explicit numerical estimates (e.g., “In 90% of cases…”), and references to frequencies were rare. Most relied on coarse-grained terms like “might be,” “hard to say,” or hybrid formulations such as “Could be, but not necessarily.” In response to “A person does sport. Does she lose weight?”, one participant wrote: “Could be, but doesn’t have to” (German original: „Könnte sein, muss aber nicht”). Another stated: “Not necessarily, but with a certain probability” („Nicht unbedingt, aber mit einer gewissen Wahrscheinlichkeit”).

Finally, some participants’ responses combined both forms of uncertainty – invoking probabilistic language while also referencing possible exceptions. For instance, in response to the conditional “If a person is called, does their phone ring?”, one participant wrote: “Yes, probably, unless it’s on silent – most of the time it rings.” Since these responses clearly rely on counterexamples, they were classified accordingly, as the probabilistic terms were evidently used only as a generic expression of uncertainty. Overall, counterexample-based reasoning emerged as the most prevalent and cognitively salient form of explanation, aligning well with our quantitative finding.

## General discussion

The methods employed in our experiments were deliberately designed to minimize external constraints on participants’ reasoning and to capture their conclusions in a form that is as spontaneous and natural as possible. In this respect, the approach shares some features with introspective methods in psychology. It is well established, however, that people have limited introspective access to their own cognitive processes and may instead rely on implicit theories or post hoc rationalizations. Moreover, prompting individuals to reflect on their reasoning can itself alter the reasoning process (e.g., Nisbett & Wilson, 1977). Nonetheless, we believe that, when interpreted with appropriate caution, our findings can offer valuable insights, in particular as our study does not abandon experimental rigor. In fact, our method remains firmly within the experimental paradigm. It departs from conventional protocols in only one key respect: instead of restricting participants to pre-defined response categories or rating scales, it allows them to articulate their reasoning in their own words, showing that introspective approaches can effectively complement standard experimental designs.

Our first hypothesis concerned the frequency of certain responses in conditional reasoning. Strikingly, nearly 30% of all responses in our open-ended format were confident and categorical – even in scenarios that lacked a formal logical structure. This finding directly challenges a foundational assumption of probabilistic theories: namely, that people predominantly interpret conditionals as uncertain and inherently gradable.

Much of the empirical support for this probabilistic approach stems from studies that rely on rating scales. However, maybe these formats inherently steer participants toward expressing uncertainty, both by framing the task probabilistically and by discouraging binary or categorical thinking. By contrast, our use of open-response formats allowed participants to formulate their reasoning without such constraints. The result was a marked increase in categorical judgments and logically structured explanations – suggesting that deductive reasoning is a psychologically accessible and frequently applied strategy, especially in the absence of uncertainty priming.

This methodological contrast reveals an important insight: what appears as probabilistic reasoning may often be a product of the measurement tool itself. We propose the term *probabilistic masking* to describe this phenomenon – where standard rating formats induce probabilistic inferences that mask the presence of alternative, often deductive, reasoning strategies. That is, the very act of asking for a graded response may suppress the expression of counterexamples, nudge people toward uncertainty, and thus lead to an overrepresentation of probabilistic interpretations in the data. Open formats, by contrast, reveal a richer repertoire of reasoning strategies – including the spontaneous generation of counterexamples, the articulation of exception conditions, and the use of deductive schemata – often hidden beneath the surface of conventional response scales. Note that this is exactly the opposite of the claim made by probability theorists that deductive inferences are in fact probabilistic inferences involving extreme probabilities (Oksford, 1995).

Our second hypothesis concerned the way people explain their uncertainty. Across both experiments, participants most frequently reasoned via counterexamples. This pattern emerged clearly in both our quantitative analyses and the qualitative inspection of participants’ answers. When participants rejected a conclusion, they often justified their uncertainty by describing situations in which p is true, but q does not follow – frequently in the form of vivid, concrete scenarios.

This naturally raises the question of whether the number of counterexamples cited influences the degree of uncertainty. Unfortunately, our data do not allow us to answer this question conclusively. Previous research has offered competing hypotheses on this matter. While Markovits et al. (2010) suggest that uncertainty emerges once a threshold number of counterexamples is reached, others propose a more linear relationship between the number of counterexamples and the perceived credibility of the conclusion (De Neys, et al., 2003, 2005). Interestingly, our participants were not required to produce counterexamples at all – yet many did, sometimes providing one, sometimes two or three, and rarely more.

This finding gains additional importance when considered in light of our methodological approach. We took particular care to design our coding scheme in a way that would not favor either mental model theory or the probabilistic account. In retrospect, however, we suspect that our method may have underestimated the prevalence of counterexample-based reasoning. Specifically, we classified any response as “probabilistic” if it included hedging expressions such as “probably,” “possibly,” or “maybe,” whenever no explicit counterexample was given. But it is entirely plausible that participants used such expressions while still mentally relying on counterexamples they simply did not articulate. Similarly, some participants may have mentioned only one counterexample, despite having others in mind. Writing down multiple counterexamples is demanding, and it would be unrealistic to assume that participants were always motivated to do so. Taken together, this means that many responses we categorized as “probabilistic” may, in fact, have been based on unarticulated counterexamples.

Nonetheless, the fact that more responses featured explicit counterexamples than probabilistic expressions lends strong support to the mental model theory. Participants clearly considered alternative possibilities to evaluate the plausibility of conclusions. The results also shed new light on the cognitive processes underlying the suppression effect (Byrne, 1989). Even though no exceptional circumstances were presented, suggested, or required by the task, participants spontaneously considered such possibilities. This, in turn, reduced the perceived plausibility of otherwise logically valid conclusions. Equally noteworthy is that participants often seemed aware of cases where not-p but q holds, leading to less AC errors than in other studies.

At the same time, our findings pose a challenge to probabilistic accounts of reasoning. While we did observe some responses framed in terms of likelihood, these were in the minority. Given the relatively “soft” nature of our coding criteria, we refrain from overinterpreting this result. Still, we invite proponents of probabilistic models to engage with our findings. Is it not striking that participants so often used counterexamples and so rarely employed probabilistic language when explaining their reasoning?

Our results align with recent arguments suggesting that method is never theory-neutral (Lejarraga & Hertwig, 2021). Even subtle methodological choices can shape outcomes in significant ways – a point we have observed in many of our past experiments. For instance, in previous studies we have shown that seemingly minimal manipulations – such as using an abstract noun versus a proper name, or verbal framing can lead to significant differences in reasoning outcomes (Knauff & Gazzo Castañeda, 2016a, 2016b, 2018, 2019, 2022). Moreover, neuroimaging studies from our lab have shown that participants are capable of flexibly switching between different reasoning strategies, depending on task demands. Importantly, we have also found consistent activation in brain regions typically associated with logical reasoning, suggesting that deductive processes remain a relevant and robust component of human thought (Gazzo Castaneda, et al., 2023).

Taken together, our findings reinforce our call for a more cooperative and theory-integrative approach to the study of reasoning – an approach that has been referred to as *adversarial collaboration* (Mellers et al., 2001). In Knauff and Gazzo Castañeda (2021, 2023), we have argued for greater cooperation between theoretical frameworks – particularly through the collaborative design of empirical studies that avoid nudging participants toward predefined answers or reasoning strategies. Task format is a theory and open responses, as used in our study, may help uncover a broader repertoire of reasoning strategies that often remain hidden in tasks with restricted response options. We see our study as a valuable starting point for further research along these lines.

## Electronic supplementary material

Below is the link to the electronic supplementary material.

Supplementary file1 (DOCX 42 KB)

## Data Availability

All data are available online (https://osf.io/y4689/). Materials are provided in the *Methods* section. None of the experiments were preregistered.

## Appendix

The following examples illustrate participants’ open-ended responses to two of the conditional reasoning tasks from both experiments. The examples are grouped by response type: (1) uncertain responses justified by counterexamples, (2) uncertain responses using probabilistic expressions. Certain responses (e.g., “Yes”, “No”) are not presented, as they typically lacked explanatory detail and were uninformative. All responses have been translated from German and slightly edited for clarity while preserving their original content and intent. The full materials and all particiants’ responses can be found on https://osf.io/y4689

**Table:**

**If a person is called, then her phone rings**	**If a person exercises, then she loses weight**
**Answers using Counterexamples** **MP, affirmative** Yes, if it is not on silent. Yes, unless it is muted or turned off. Yes, or it vibrates. If the phone is not on silent, yes. Yes, if it does not vibrate. Only if it is on loud. **MP, negative:** No, the phone could be set to silent or vibrate. No, or it is an alarm clock. It could be that the person is being called, but it could also be an alarm clock ringing. Not necessarily, the person might have set the phone to silent. If it is turned off, then no. No, only if it is set to loud. Not necessarily, it could be turned off or in a dead zone. **AC, affirmative:** Yes, or it is an alarm or a reminder. Very likely, but someone may have misdialed. **AC, negative:** No. Could also be a message. No, not necessarily, it could also be her alarm clock ringing. **Answers using probabilities** **MP, affirmative:** Presumably Probably yes Normally, yes.	**Answers using Counterexamples** **MP, affirmative:** Yes, if they watch their diet. Yes, but only if they are in a calorie deficit. If they consume only the maximum calories necessary for them, they can lose weight. Depending on the type of sport, however, they may also build muscle and thus gain weight. Yes, if they do a lot of sports and eat little; otherwise, it does not have to be the case that they lose weight. If they do it properly and also watch their diet. **MP, negative:** No, if the diet is not right, then not. No. They are probably building muscle, muscles are heavier. No, it also depends on how the person eats and what their goal is with exercising. Every body is different. And muscles even weigh more than fat, so this does not necessarily have to happen. Depending on the type of training, a person loses or gains weight. To lose weight, many things must be considered: regular exercise, movement, healthy diet, and genes also play a role. It also depends on the type of sport. After all, there is also e-sports. It depends on metabolism, body type, and weight. Unfortunately not necessarily. Diet, disposition, and type of sport are additional factors. **AC, affirmative:** Either that or they are ill (if it’s a very strong weight loss). Could be, but could also be that they are starving or ill. Possible, but could also be a diet. Yes and/or they eat healthily. **AC, negative:** No, there are also other methods to lose weight. She could also have cancer. Not necessarily. One loses weight through a calorie deficit. One can also gain weight through exercise. Weight loss can occur for various reasons. Exercise is one possibility, but there are many other factors, e.g. diet, illnesses, etc. Not necessarily. Also possible through fasting or a change in diet. Not necessarily. Maybe the person just watches their calorie intake. No. There are many reasons to lose weight. Sport would be one possibility, but there are unfortunately also many illnesses, eating disorders where people lose weight unintentionally, even without exercise. **Answers using Probabilities** **MP, affirmative:** Sport or a diet, with sport being very helpful for losing weight – so presumably. If a person exercises, then they can lose weight. **AC, affirmative:** You can lose weight if you exercise. Yes, most likely. Could be, but doesn’t have to be. It can contribute to it.

## Declarations

### Open practices statement

All data are available online (https://osf.io/y4689/). None of the experiments were preregistered.

nnnn
