# Supplementary material for: Reasoning beyond clicks: Disentangling counterexamples and probabilities in conditional reasoning
Source: Mem Cognit. 2025 Nov 4;54(3):1052–63. doi: 10.3758/s13421-025-01796-9 (PMC13132893; doi:10.3758/s13421-025-01796-9)
Supplement: Supplementary file 1 — Supplementary file1 (DOCX 42 KB) [file 13421_2025_1796_MOESM1_ESM.docx]

**Appendix**

The following examples illustrate participants’ open-ended responses to two of the conditional reasoning tasks from both experiments. The examples are grouped by response type: (1) uncertain responses justified by counterexamples, (2) uncertain responses using probabilistic expressions. Certain responses (e.g., “Yes,” “No”) are not presented, as they typically lacked explanatory detail and were uninformative. All responses have been translated from German and slightly edited for clarity while preserving their original content and intent. The full materials and all particiants’ responses can be found on the Open Science Framework at: <https://osf.io/y4689>

| **If a person is called, then her phone rings** | **If a person exercises, then she loses weight** |
| --- | --- |
| **Answers using Counterexamples**  **MP, affirmative**  Yes, if it is not on silent.  Yes, unless it is muted or turned off.  Yes, or it vibrates.  If the phone is not on silent, yes.  Yes, if it does not vibrate.  Only if it is on loud.  **MP, negative:**  No, the phone could be set to silent or vibrate.  No, or it is an alarm clock.  It could be that the person is being called, but it could also be an alarm clock ringing.  Not necessarily, the person might have set the phone to silent.  If it is turned off, then no.  No, only if it is set to loud.  Not necessarily, it could be turned off or in a dead zone.  **AC, affirmative:**  Yes, or it is an alarm or a reminder.  Very likely, but someone may have misdialed.  **AC, negative:**  No. Could also be a message.  No, not necessarily, it could also be her alarm clock ringing.  **Answers using probabilities**  **MP, affirmative:**  Presumably  Probably yes  Normally, yes. | **Answers using Counterexamples**  **MP, affirmative:**  Yes, if they watch their diet.  Yes, but only if they are in a calorie deficit.  If they consume only the maximum calories necessary for them, they can lose weight. Depending on the type of sport, however, they may also build muscle and thus gain weight.  Yes, if they do a lot of sports and eat little; otherwise, it does not have to be the case that they lose weight.  If they do it properly and also watch their diet.  **MP, negative:**  No, if the diet is not right, then not.  No. They are probably building muscle, muscles are heavier.  No, it also depends on how the person eats and what their goal is with exercising.  Everybody is different. And muscles even weigh more than fat, so this does not necessarily have to happen. Depending on the type of training, a person loses or gains weight.  To lose weight, many things must be considered: regular exercise, movement, healthy diet, and genes also play a role.  It also depends on the type of sport. After all, there is also e-sports.  It depends on metabolism, body type, and weight.  Unfortunately, not necessarily. Diet, disposition, and type of sport are additional factors.  **AC, affirmative:**  Either that or they are ill (if it’s a very strong weight loss).  Could be, but could also be that they are starving or ill.  Possible, but could also be a diet.  Yes and/or they eat healthily.  **AC, negative:**  No, there are also other methods to lose weight.  She could also have cancer.  Not necessarily. One loses weight through a calorie deficit. One can also gain weight through exercise.  Weight loss can occur for various reasons. Exercise is one possibility, but there are many other factors, e.g. diet, illnesses, etc.  Not necessarily. Also possible through fasting or a change in diet.  Not necessarily. Maybe the person just watches their calorie intake.  No. There are many reasons to lose weight. Sport would be one possibility, but there are unfortunately also many illnesses, eating disorders where people lose weight unintentionally, even without exercise.  **Answers using Probabilities**  **MP, affirmative:**  Sport or a diet, with sport being very helpful for losing weight – so presumably.  If a person exercises, then they can lose weight.  **AC, affirmative:**  You can lose weight if you exercise.  Yes, most likely.  Could be, but doesn’t have to be.  It can contribute to it. |
